# A CGRP receptor antagonist peptide formulated for nasal administration to treat migraine

**DOI:** 10.1111/jphp.13317

**Published:** 2020-06-25

**Authors:** Bengt von Mentzer, Andrew F Russo, Zhongming Zhang, Adisa Kuburas, Patrick M Killoran, Vera D’Aloisio, Laura Nizic, Vicky Capel, David A Kendall, Christopher R Coxon, Gillian A Hutcheon

**Affiliations:** Innovipharm Limited, West Kirby, UK; Department of Molecular Physiology and Biophysics, Center for Prevention and Treatment of Visual Loss, Veterans Administration Health Center, University of Iowa, Iowa City, IA, USA; College of Medicine, Henan Key Laboratory of Zhang Zhongjing Formulae and Herbs for Immunoregulation, Nanyang Institute of Technology, Nanyang, Henan, China; Division of Structural Biology (STRUBI), Harwell Campus, University of Oxford, Didcot, UK; School of Pharmacy and Biomolecular Sciences, Liverpool John Moores University, Liverpool, UK; Faculty of Pharmacy and Biochemistry, University of Zagreb, Zagreb, Croatia; Catalent Pharma Solutions, Nottingham, UK; Institute of Chemical Sciences, School of Engineering and Physical Sciences, Heriot-Watt University, Edinburgh, UK

**Keywords:** aCGRP (8-37), aCGRP (27-37), cAMP, plasma extravasation, SK-N-MC

## Abstract

**Objectives:**

To investigate the formulation of the peptide-based antagonist (^34^Pro,^35^Phe)CGRP_27–37_, of the human calcitonin gene-related peptide (CGRP) receptor as a potential nasally delivered migraine treatment.

**Methods:**

Peptide sequences were prepared using automated methods and purified by preparative HPLC. Their structure and stability were determined by LC-MS. Antagonist potency was assessed by measuring CGRP-stimulated cAMP accumulation in SK-N-MC, cells and in CHO cells overexpressing the human CGRP receptor. *In vivo* activity was tested in plasma protein extravasation (PPE) studies using Evans blue dye accumulation. Peptide-containing chitosan microparticles were prepared by spray drying.

**Key findings:**

(^34^Pro,^35^Phe)CGRP_27–37_ exhibited a 10-fold increased affinity compared to αCGRP_27–37_. Administration of (^34^Pro,^35^Phe)CGRP_27–37_ to mice led to a significant decrease in CGRP-induced PPE confirming antagonistic properties *in vivo*. There was no degradation of (^34^Pro,^35^Phe)CGRP_27–37_ and no loss of antagonist potency during formulation and release from chitosan microparticles.

**Conclusions:**

(^34^Pro,^35^Phe)CGRP_27–37_ is a potent CGRP receptor antagonist both *in vitro* and *in vivo,* and it can be formulated as a dry powder with no loss of activity indicating its potential as a nasally formulated anti-migraine medicine.

## Introduction

Migraine is a common, frequently chronic, neurovascular disorder of the brain characterised by recurrent, severe attacks of headache often associated with nausea and sensory hypersensitivity rendering suffers unable to function at work or at home.^[1]^ This has a severe negative impact on the individual patient, their family and society.^[2]^ Migraine is the third most common disease in the world (behind dental caries and tension-type headache) with an global prevalence of over 14% and with woman three times more likely to be the sufferer.^[3]^

While the mechanisms underlying migraine remain relatively elusive, neurochemical and pharmacological observations have established the functional importance of the neuropeptide calcitonin gene-related peptide (CGRP) in the pathogenesis of migraine.^[4,30]^ Infusion of CGRP causes delayed headaches in migraineurs^[5,6]^ and increased levels of CGRP are found in the serum and saliva of patients during attacks.^[7]^ Despite the limitations of animal models of migraine, humanised transgenic mice overexpressing CGRP receptors exhibit photophobic behaviour similar to that observed during a migraine attack and these mice exhibit an increased CGRP-induced mechanical allodynia, a phenomenon also common in migraineurs.^[8]^ Most importantly from a drug development perspective, a number of ‘gepants’, small molecule CGRP receptor antagonists, (olcegepant, telcagepant, MK-3207, BI 44370 TA and BMS-927711) entered clinical trials and some demonstrated clinical efficacy despite having drawbacks related to hepatotoxicity or poor pharmacokinetics.^[9]^ In December 2019, ubrogepant was FDA approved as the first small molecule CGRP antagonist for migraine. A different approach to small molecule antagonist development has been to produce humanised monoclonal antibodies (mAbs) to CGRP or the CGRP receptor. Encouraging results have been observed in trials with a significant number of participants experiencing complete relief from migraine pain.^[10]^ In 2018, Aimovig (Amgen/Novartis) was the first to market a mAb migraine prevention drug and was closely followed by Ajovy (Teva) and Emgality (Eli Lilly) and most recently Vyepti (Lundbeck Seattle Biopharmaceuticals).

However, CGRP is a vasodilatory safeguard against cerebral and cardiac ischemia, and there are some concerns about the risks of continuous CGRP blockade provided by mAb injection.^[29]^

An alternative anti-migraine strategy is the development of peptide-based CGRP receptor antagonists. Truncated C-terminal amidated fragments of CGRP, for example CGRP_8-37_,^[11,12]^ CGRP_27-37_ and various other analogues^[13]^ are known to act as antagonists of the CGRP receptor (Table 1).

**Table 1:** The sequences of full length native human α-CGRP and N-terminally truncated analogues that act as antagonists of the CGRP receptor

	1	2		3	4	5	6	7	8	9	10	11	12	13	14	15	16	17	18	19	20	21	22	23	24	25	26	27	28	29	30	31	32	33	34	35	36	37	38
αCGRP_1–37_	A	C^a^		D	T	A	T	C^a^	V	T	H	R	L	A	G	L	L	S	R	S	G	G	V	V	K	N	N	F	V	P	T	N	V	G	S	K	A	F	NH_2_
αCGRP_8–37_									V	T	H	R	L	A	G	L	L	S	R	S	G	G	V	V	K	N	N	F	V	P	T	N	V	G	S	K	A	F	NH_2_
αCGRP_27–37_																												F	V	P	T	N	V	G	S	K	A	F	NH_2_
(^34^Pro,^35^Phe)CGRP_27–37_																												F	V	P	T	N	V	G	**P**	**F**	A	F	NH_2_

**P** and **F** are the amino acids substituted into the truncated CGRP_27-37_ to produce the (^34^Pro,^35^Phe)CGRP_27–37_ analogue.

a Sites of disulphide bridges.

Unlike many small organic molecules, small peptides are much less likely to produce potentially toxic hepatic metabolites or to be immunogenic.^[14]^ In addition, from a manufacturing point of view, mAbs are more expensive to produce,^[15]^ whereas small peptides are generally easier, lower cost and also allow better control of homogeneity.

However, a major challenge in relation to the use of peptides as medicines is their limited absorption and rapid metabolism in vivo leading to poor bioavailability.^[16]^ Subcutaneous injection is often employed but can lead to poor patient compliance and injection site reactions. There has been increasing interest in nose to brain delivery due to the perception that the nasal mucosa is a less demanding physiological barrier than other routes of administration.^[17,18]^ Intranasal delivery involves both the olfactory and trigeminal pathways, generally lower doses can be used and the dosage volume is small but exact dosing can be a challenge.^[32]^

Additionally, increased patient compliance, improved safety and a rapid onset of action are all features of nasal delivery.^[33]^

Recent developments in the delivery of drugs to the brain, including the use of delivery vehicles such as micro- and nano-carriers alongside mucoadhesive agents such as chitosan, are promising.^[19]^

In this study, we have investigated the antagonist properties of a synthetic N-terminally truncated and modified human CGRP peptide. We show that (^34^Pro,^35^Phe)CGRP_27–37_ is a potent CGRP receptor antagonist both in vitro and, for the first time, in vivo and demonstrate how it can be formulated for dry powder nasal delivery without loss of potency.

## Materials and Methods

### Materials

Human αCGRP and CGRP analogues (Bachem AG, Budendorf, Switzerland) were dissolved in pure water and kept frozen (−18 °C) in aliquots before use.

All Fmoc l- and d-amino acids (CEM Microwave Technology Ltd, Buckingham, UK), Rink Amide ProTide resin (CEM), diisopropylcarbodiimide (DIC; Apollo Scientific, Stockport, UK), Oxyma Pure (CEM), N.N′-dimethylformamide (DMF; Fisher Scientific, Loughborough, UK), diisopropylethylamine (DIPEA; Merck Millipore, Watford, UK) and piperidine (Merck Millipore) were purchased from commercial suppliers and used directly as indicated in the appropriate experimental procedures. SK-N-MC cells were purchased from ATCC (Manassas, VA, USA). Earle’s Balanced Salts, l-glutamine, fetal bovine serum (FBS), sodium pyruvate and non-essential amino acids were purchased from Life Technologies (Stockholm, Sweden). HitHunter cyclic AMP assay kits were purchased from DiscoveRx (Eurofins, Fremont, CA, USA). All other reagents (trifluoroacetic acid (TFA), triisopropylsilane (TIPS), chitosan [CAS number 9012-76-4]) were purchased from Sigma-Aldrich (Gillingham, UK), and solvents (HPLC grade) were purchased from Fisher Scientific (Loughborough, UK).

### Peptide synthesis

Peptide sequences were prepared as C-terminal amides using automated Fmoc-SPPS methods on a Liberty Blue microwave-assisted peptide synthesiser (CEM). Solid-phase synthesis was conducted using Rink amide ProTide resin (180 mg, 0.56 mmol/g loading; 0.1 mmol), employing the required Fmoc amino acids (0.2 m in DMF; 5 eq.); with DIC (1 m stock solution in DMF; 10 eq.), Oxyma Pure (1 m stock solution, 5 eq) and piperidine (20% *v/v* in DMF; 587 eq., 4 ml) as activator, and deprotection, respectively. Standard coupling procedures employed double coupling of each amino acid (2.5 min, 90 °C). Finally, peptides were cleaved from the resin as the C-terminal amide by treatment with a cleavage cocktail (5 ml; comprising TFA, TIPS and water (9: 0.5: 0.5 *v/v*) with regular shaking at room temperature for 4 h. Peptides were precipitated from cleavage solutions by dropwise addition into cold diethyl ether followed by centrifugation. The resulting pellet was successively suspended in cold diethyl ether and centrifuged twice further. The solids obtained were dissolved in H_2_O/MeCN (depending upon solubility), frozen, lyophilised and purified by semi-preparative HPLC.

#### HPLC

Analytical and semi-preparative HPLC employed an Agilent 1200 Series HPLC comprising a diode-array detector (215/280 nm) and G1364C fraction collector (semi-prep only).

#### Analytical HPLC

Peptides were solubilised in MeCN and H_2_O and separated using a Phenomenex C18 analytical HPLC column (3.6 μm particle size, 4.6 × 150 mm column) with a binary eluent system comprising MeOH/H_2_O (18 min gradient: 5–95% with 0.1% formic acid) as mobile phase. Operating pressures were in the range of 2000–3000 PSI.

#### Semi-preparative HPLC purification of peptides

Crude samples (25 mg/ml; 40 μl injection) were separated using a XBridge Peptide BEH C18 Prep 130 Å 5 μm column (10 × 150 mm) (Waters) with a binary eluent system comprising MeOH and H_2_O (with 0.1% v/v formic acid) as mobile phase. Operating pressures were around 2000 PSI. Isolated pure peptides were concentrated *in vacuo* to remove organic volatiles, and the aqueous solutions were then flash-frozen (liquid N_2_) and lyophilised.

#### Mass spectrometry

Samples were analysed using an Agilent 1260 Infinity II LC system with Agilent 6530 Accurate-Mass QToF spectrometer, using an Agilent ZORBAX Eclipse Plus C18 Rapid Resolution HD analytical column (1.8 μm particle size, 2.1 × 50 mm) with a binary eluent system comprising MeOH/H_2_O (12 min gradient: 1–99% with 0.1% formic acid) as mobile phase. Operating pressures were in the range of 2000–3000 PSI. Electrospray ionisation mass spectrometry was conducted in positive ion mode (*m/z* range: 50–3200) using a fragmentor voltage of 150 V, gas temperature of 325 °C (flow 10 l/min) and sheath gas temperature of 400 °C (flow 11 l/min). All peptides were dissolved in pure water and kept frozen (−18 °C) in aliquots before use.

### Cyclic AMP accumulation

SK-N-MC cells (neuroblastoma cell line of human origin, ATCC) were aliquoted (10^7^ cells/ml) and frozen (−150 °C) in a mixture of 10 % DMSO in culture medium consisting of minimum essential medium (MEM), with Earle’s salts, l-glutamine, 10 % FBS, 1 % sodium pyruvate and 1% non-essential amino acids. Cells were rapidly thawed in 37 °C, centrifuged (300 *g*, 5 min) and suspended in culture medium (37 °C) before seeding in 175 cm^2^ flasks (Nunclon Δ surface) in an atmosphere containing 5 % CO_2_ at 37 °C for 2–3 days. The cell cultures were used at 80% confluency. The culture medium was removed and washed in 10 ml PBS Dulbecco’s medium without sodium bicarbonate. The cells were detached by adding 10 ml PBS Dulbecco’s without calcium, magnesium and sodium bicarbonate supplemented with 1 mm EDTA. The cell suspension was centrifuged (300 *g*, 5 min), and the pellet suspended in 5 ml culture medium. 5 million cells were seeded into 175 cm^2^ flasks in 40 ml 37 °C culture medium. Each batch of cells was kept for 6–7 passages only.

In cyclic AMP (cAMP) experiments, 40 000 cells in 250 µl culture medium were added to each well (3595 Costar) and cultured for 24–28 h to obtain a confluent layer of cells. Each experiment was initiated by removing the medium and blotting on a piece of paper tissue. Each well was gently washed twice with 200 µl cAMP buffer containing 2.5 mm Tris, 2.5 mm HEPES, 140 mm NaCl, 5 mm KCl, 1.8 mm CaCl_2_, 4.5 g/l glucose, 0.2 % bovine serum albumin (BSA) and 0.1 mm isobutyl methyl xanthine, pH 7.4 at 20 °C followed by the blotting procedure and 240 µl cAMP buffer was added to each well. The plate was pre-incubated for 30–60 min at 37 °C before addition of 10 µl of antagonist or plain buffer. Following 10 min pre-incubation (37 °C), 50 µl of αCGRP was added in different concentrations and CGRP receptor stimulation continued for 20 min. The reaction was interrupted by quickly aspirating off the reaction mixture, and 200 µl acidified methanol, pH 2, was added to lyse the cells. 150 µl of the mixture was transferred from each well to a new 96-well plate, and the methanol was totally evaporated at 50 °C. The plate was thereafter kept frozen at −18 °C for later analysis of cAMP by an enzyme immunoassay (RPN 225, Amersham Pharmacia Biotech, UK) following the non-acetylation assay. 250 µl assay buffer was added to each well of the plate from the cAMP experiment; 100 µl samples from each well and 100 µl of standard solutions were transferred to a 96-well micro-titre plate. The optical density was determined in a microplate reader (Thermo max, Molecular Devices) at 450 nm and cAMP content calculated by reference to the standard curve.

For some experiments, CGRP-stimulated cAMP accumulation was assayed in CHO cells overexpressing the human CGRP receptor complex (receptor activity-modifying protein-1 (RAMP1) and calcitonin receptor-like receptor (CLR)) using the commercial DiscoveRx HitHunter assay kit. Increasing concentrations (0.01–30 nm) of αCGRP were added to the cells, and the agonist EC_50_ values determined in the presence of different concentrations of antagonist peptide (30, 100, 300 nm or 1 μm). *K*_B_ values (antagonist affinity constant) were obtained from Shild analysis.^[20,21]^ Log (conc. ratio-1) was plotted against log [antagonist conc.], and log *K*_B_ was determined from the abscissa intercept of the linear regression line. In some experiments, *K*_B_ was estimated from shifts in CGRP concentration/response curves using a single antagonist concentration and applying the Schild equation; (agonist conc. ratio − 1) = (antagonist conc.)/*K*_B_). The concentration ratio is the agonist EC_50_ value in the presence of antagonist/EC_50_ value in the absence of antagonist. Protein concentrations of membranes and SK-N-MC cells were determined by a Bio-Rad kit (Bio-Rad, CA, USA). Lyophilised bovine plasma gamma globulin was used as protein standard (Bio-Rad).

### Plasma protein extravasation

Blood extravasation testing was performed as previously described^[22]^ with some minor modifications. Briefly, 10 mg/kg Evans blue in PBS was injected intravenously into the tail vein of four different cohorts of C57Bl/6J mice, 2–6 months of age. Mice were then anaesthetised using 72/13 mg/kg, i.p., ketamine/xylazine. For each cohort, αCGRP (20 µl of 250 nm aqueous solution; 5 pmol) was subcutaneously injected into one hind paw of anaesthetised mice. PBS or the combination of CGRP (250 nm) plus experimental peptide antagonists (CGRP_8-37_, (^34^Pro,^35^Phe)CGRP_27–37_, or CGRP_27-36_) (100 nm) was injected into the other hind paw. After 15 min, 0.2–0.4 ml blood samples were obtained by cardiac puncture and placed into EDTA collection tubes. The blood samples were centrifuged at 3000 *g* for 30 min, and the plasma samples were stored at –20 °C until analysis.

Mice were killed by CO_2_ asphyxiation after blood collection. Skin with subcutaneous tissue containing dye surrounding the injection site was excised and placed into 300 μl of extracting solution (7 : 3 mixture of acetone and 0.5% Na_2_SO_4_ solution) and pulverised to facilitate the extraction. Extraction continued overnight at room temperature, and the extract was then centrifuged at 3000 *g* for 10 min. The supernatant was transferred into a fresh tube for vacuum evaporation by Speedvac and the residue dissolved in 150 µl H_2_O. The plasma samples were diluted 100-fold with water; 50 µl of each sample was measured by absorbance at 620 nm. H_2_O served as blank and a series of diluted Evans blue solutions as a standard curve, which was linear from 0.25 to 20 µg/ml. The amount of Evans blue in the tissue was normalised to total amount of Evans blue present in plasma. The experimenters were blinded to the identity of the experimental drugs until data were analysed.

### Stability of (^34^Pro,^35^Phe)CGRP_27–37_ in human serum

Peptide stability in blood was investigated *in vitro* using human serum. Here, 10 μl of aqueous peptide stock solution (2 mg/ml) was added to pooled aqueous human serum (25% *v/v*, 190 μl) affording a final peptide concentration of 50 μg/ml. The mixtures were incubated at 37 °C, and the initial time was recorded. At known time intervals (0, 6, 10 and 30 min), three samples were precipitated by the addition of aqueous TCA solution (6% *v/v*, 200 μl). The cloudy reaction samples were cooled in ice for 15 min and then centrifuged (13 200 rpm, 2 min) (Eppendorf Centrifuge 5415 D) to pellet the precipitated serum proteins. The supernatants were analysed using RP-HPLC (described above). LC-MS analysis of the formulation supernatant was performed to ensure that the peptide was not degraded.

### Formulation

Peptide-containing microcarriers were prepared by spray drying (^34^Pro,^35^Phe)CGRP_27–37_, (1%) and low molecular weight (LWM) chitosan (2%), from a solution of 0.5% acetic acid. A Büchi B-290 spray dryer (Büchi Labortechnik AG, Postfach, Switzerland) equipped with a nozzle atomizer with a nozzle orifice diameter of 2.0 mm was used. Nitrogen was used as the atomising gas, and dry particles were separated from the airstream by centrifugal forces using a high-performance cyclone (Büchi Labortechnik AG). Taguchi experimental design was employed to determine the optimal operating parameters, constant spray gas flow (60) and feed flow rate (15% of pump capacity, just under 5 ml/min), aspirator capacity 75, 85 and 95%, and inlet temperature, 150, 165 and 180 °C. Moisture content was determined by thermogravimetric analysis by TGA Q5000 (TA Instruments, New Castle, DE, SAD). The spray-dried powder (10 mg) was loaded on a platinum TGA pan suspended from a microbalance and heated from 25 °C to 250 °C at 10 °C per min. The water loss between 25 and 100 °C was analysed.

The morphology and microparticle diameter was observed using scanning electron microscope. The average diameter of the particles was calculated with ImageJ 1.51 software measuring 100 particles for each sample. Release of the peptide from the chitosan microcarrier into deionised water was determined by suspending MPs (10 mg) in deionised water (1 ml) in a 1.5 ml centrifuge tube and mixing at 20 rpm and 37 °C, utilising the unloaded MPs as blanks. Three samples for each replicate were prepared, and at each time point (1, 4 and 24 h), one sample was centrifuged at 13 200 rpm for 10 min (Eppendorf Centrifuge 5415 D). The supernatant solution was analysed by HPLC (Agilent Poroshell 120 EC-C18 column 2.7 μm particle size 3.0 × 50 mm) using a linear gradient from 99 % solution A (high purity water 0.1% formic acid) to 100% solution B (MeCN 0.1% formic acid) over 10 min. A flow rate of 0.600 ml/min was used, the absorbance detected at 215 nm and the analysis was performed at room temperature (25 °C).

### Ethical approval

Animal procedures were approved by the University of Iowa Animal Care and Use Committee (approval #0711247) and performed in accordance with the standards set by the National Institutes of Health.

### Data analysis and statistical calculations

Results were evaluated with Excel fit or GraphPad Prism. Results were determined as means ± standard error. Statistical analysis was performed by applying Student’s *t*-test or ANOVA where appropriate.

## Results and Discussion

This investigation evaluated (^34^Pro,^35^Phe)CGRP_27–37_, a modified analogue of αCGRP (Table 1), as a potent CGRP receptor antagonist *in vitro* and *in vivo* to demonstrate its potential as a new nasal migraine medicine.

The known antagonist (^34^Pro, ^35^Phe)CGRP_27-37_, in which both ^34^Ser and ^35^Lys were replaced with proline and phenylalanine, respectively, was synthesised. As a negative control, an αCGRP_27-36_ analogue lacking the ^37^Phe residue was also prepared. All peptides were prepared as C-terminal amides on Rink Amide resin using standard solid-phase peptide synthesis protocols and purified by preparative HPLC.

**Figure 1:**
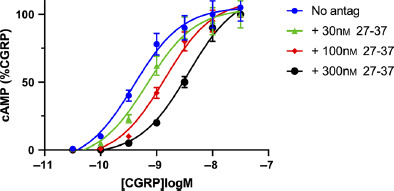
Inhibition of CGRP-stimulated cAMP accumulation. CGRP concentration/response curves in SK-N-MC cells showing the accumulation of cAMP without (^34^Pro,^35^Phe)CGRP_27-37_ and in the presence of 30 nm (•), 100 nm (▲) and 300 nm (▼) (^34^Pro, ^35^Phe) CGRP_27-37_. The Schild slope (not shown) based on the parallel shifts in the agonist concentration/response curves was 0.94 ± 0.06 with an antagonist affinity constant (*K*_B_) of 48 nm. The data are expressed as percentages of the maximal effect of CGRP alone and indicate means ± SEM (*n* = 7).

**Figure 2:**
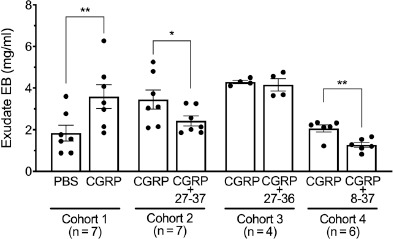
The effects of three modified CGRP peptides on CGRP-induced plasma protein extravasation in mice. For cohort 1, the vehicle PBS was injected into one hind paw and CGRP was injected into the other hind paw of the same animal. For cohorts 2-4, CGRP was injected into one hind paw and a combination of CGRP + antagonist (^34^Pro,^35^Phe)CGRP_27-37_ (27-37) (cohort 2), negative control antagonist CGRP_27-36_ (27-36) (cohort 3), positive control antagonist CGRP_8-37_ (8-37) (cohort 4) was injected into the other hind paw. In each cohort, *n* = number of mice. The level of Evans blue (EB) extravasation into the tissue was compared between the paws of the same mouse. The means ± SEM are shown, **P = 0.0039 CGRP vs PBS, *P = 0.0204 CGRP vs CGRP + 27-37, **P = 0.0011 CGRP vs CGRP + 8-37.

αCGRP increased cAMP accumulation in a concentration-related fashion with an EC_50_ of 0.4 ± 0.03 nm (SK-N-MC cells) and 0.1 ± 0.01 nm (DiscoveRx assay). Increasing concentrations of CGRP_8-37_ produced parallel rightwards shifts in the CGRP concentration/response curves with no change in maximum responses, indicating a competitive antagonism (Figure 1). Schild analysis revealed an antagonist potency (*K*_B_) of 158 ± 0.2 nm (*n* = 7). CGRP_8-37_ had a *K*_B_ of 794 ± 20 nm (*n* = 7) and (^34^Pro,^35^Phe)CGRP_27-37_ demonstrated a 10-fold enhanced potency (*K*_B_ = 79 ± 0.8 nm; *n* = 7). Activation of adenylyl cyclase and increased accumulation of cAMP are the major signal transduction pathways for the CGRP receptor complex, and these data are consistent with (^34^Pro,^35^Phe)CGRP_27-37_ acting as a potent functional antagonist with the potential to block the effects of endogenous CGRP and, thereby, to have an anti-migraine action.^[4]^ αCGRP-induced plasma protein extravasation (PPE) in C57Bl/6J mice was employed as a measure of *in vivo* antagonism of CGRP receptor function (Figure 2). This assay was an *in vivo* assessment of CGRP activity and not an assay of a migraine symptom. Indeed, while it is doubtful that plasma extravasation plays a role in migraine^[31]^ it does provide a means to monitor exogenous CGRP activity. The expected outcome was that subdural injection of CGRP would cause PPE in the hind paw, which can be measured by leakage of Evans blue dye from blood vessels into the tissue. A useful feature of this assay is that extravasation can be compared between two paws in the same mouse; hence, each mouse had an internal control. The optimal αCGRP dose of 5 pmol was estimated from the literature^[28]^ and confirmed by empirical testing. This dose of αCGRP doubled the accumulation of dye in the hind paw (Cohort 1) compared to vehicle (PBS) control. In three other cohorts of mice, extravasation was compared between CGRP versus CGRP plus antagonist. The CGRP-induced increase was completely blocked with (^34^Pro,^35^Phe)CGRP_27-37_ (Cohort 2). As a negative control, the modified CGRP_27-36_ peptide was ineffective in blocking CGRP-induced extravasation (Cohort 3) and as a positive control, the known antagonist CGRP_8-37_ blocked CGRP actions (Cohort 4) (Figure 2). Rodents have previously been regarded as an inadequate model for the development of CGRP antagonists for humans, at least with regard to small molecules. For example, BIBN 4096, the first selective non-peptide CGRP antagonist, showed a 200-fold higher affinity for primate compared to rat CGRP receptors and demonstrated a remarkable affinity and selectivity for the human CGRP receptor.^[^^]^ However, it is still feasible to employ mouse models for the study of CGRP antagonism since it is possible to humanise the murine CGRP receptor complex by overexpressing the human RAMP1.^[22]^

(^34^Pro,^35^Phe)CGRP_27–37_ has previously been shown to bind with high affinity to CGRP receptors in human neuroblastoma SK-N-MC cells and to antagonise CGRP-stimulated cAMP formation.^[14]^ Our data presented here support these previous findings and provide further evidence of the enhanced potency produced by the ^34^Pro^35^Phe modification to the CGRP_27-37_ sequence.

**Figure 3:**
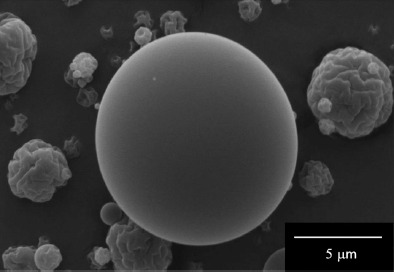
Scanning electron micrograph of peptide-loaded chitosan microparticles.

**Figure 4:**
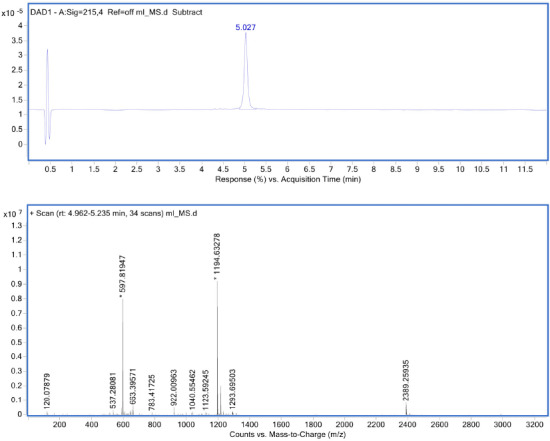
Electrospray ionisation (positive ion) mass spectrum of (^34^Pro, ^35^Phe)CGRP_27-37_ to demonstrate there was no change in peptide structure after formulation and release from chitosan nanoparticles. HPLC-MS analysis of a solution (1 mg/ml) of released peptide.

On the basis of these data, (^34^Pro,^35^Phe)CGRP_27–37_ was selected for formulation as a dry powder nasal delivery system. Formulation of peptides can be challenging,^[25]^ and the methods used are very dependent on the individual peptide physicochemical properties. A chitosan carrier was selected due to the mucoadhesive properties that would enable retention of the delivery system within the nasal cavity during peptide release. Spray-drying parameters were optimised to obtain microparticles of diameter >10 μm, initially using chitosan alone and then incorporating (^34^Pro,^35^Phe)CGRP_27–37_. Particles >10 μm are known to deposit effectively in the nasal cavity whereas particles <10 μm would be more likely to enter into the lungs. Chitosan microparticles containing 5 mg of (^34^Pro,^35^Phe)CGRP_27–37_ in 0.5 g of LMW Chitosan were prepared with a yield of 45%, which is typical for these formulations that have a tendency to stick to the walls of the cyclone. A 1% loading in chitosan as a dry powder was achieved. A final water content of 8.2% was obtained which is due to the hydrophilic nature of the chitosan.^[26]^ Scanning electron microscopy indicated that microparticles were spherical with an average diameter of 10.7 ± 1.36 μm (Figure 3). A visual examination indicated particles of variable surface roughness, some with a rough and wrinkled surface and others with a smooth surface, consistent with previous reports^[26]^ (Figure 3). Over 24 h, 70% of the (^34^Pro, ^35^Phe)CGRP_27-37_ was released into water with LC-MS indicating that the peptide structure was still intact (Figure 4). The nature of the LC-MS analysis meant that release into phosphate buffer could not be performed.

**Figure 5:**
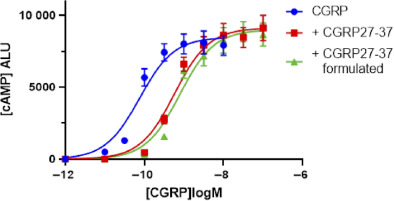
No effect of formulation on antagonist potency. CGRP-stimulated cAMP accumulation was measured in hCGRP receptor-transfected CHO cells (DiscoveRx HitHunter assay) in the absence and presence of unformulated and formulated Pro^34^Phe^35^CGRP_27-37_.cAMP accumulation is shown as mean (±SEM (*n* = 3)) arbitrary fluorescence units (ALU). The calculated antagonist potency of the peptide (130 ± 52 nm) was unaffected by formulation (126 ± 23 nm).

Analysis of the stability of the peptide in human serum showed that over a period of 30 min there was a decrease in peptide concentration from 38.3 to 13.8 μg/ml, indicating that 43 ± 15% of the peptide degraded over this time. These data show that the peptide did not degrade during formulation or release into water but, as expected, did degrade upon exposure to peptidase enzymes in the serum. Peptides typically have very short half-lives *in vivo* due to the labile bonds between amino acids so degradation of almost 50% in half an hour is typical,^[27]^ and it is anticipated that *in vivo* the rate of degradation would be much greater. Although, using a dry powder nasal delivery system is likely to reduce peptide degradation compared to parenteral delivery of a solution. The calculated antagonist potency of the peptide (130 ± 52 nm) as measured using the DiscoveRx cAMP assay was also unaffected by formulation (126 ± 23 nm) (Figure 5). The antagonist potencies calculated in the hCGRP receptor overexpressing CHO cells was somewhat lower than previously measured in the constitutively expressing SK-N-MC cells but this is not surprising given the differences in levels of receptor expression.

Future research will investigate whether further modification to the peptide structure can enhance stability or antagonist activity so that efficacy can be maintained via a smaller active-peptide dose.

## Conclusion

This investigation confirms that (^34^Pro,^35^Phe)CGRP_27–37_, a modified analogue of αCGRP, is a potent CGRP receptor antagonist and demonstrate that it could be formulated as a chitosan microparticle suitable for dry powder nasal delivery with no degradation of the peptide structure and no loss of antagonist potency upon encapsulation and release *in vitro*. We also demonstrate that the modified peptide can inhibit CGRP-enhanced PPE in the mouse.^[23]^ Proof of the concept of CGRP antagonism as a migraine therapy has been clinically demonstrated with the efficacy of monoclonal antibodies, and this study indicates the potential of nasally administered small peptides as a feasible, cost-effective and patient-approved alternative without the cardiovascular risks of continuous CGRP blockade.

## Declarations

### Conflict of interest

AFR is a consultant for Alder Lundbeck Pharmaceuticals, Eli Lilly, Amgen, Novartis, Pharmnovo and Schedule One Therapeutics. DAK and BvM are Directors of Innovipharm Ltd.

### Authors’ contributions

Capel, Coxon, Hutcheon, Kendall, Killoran and Metzer participated in research design. D’Aloisio, Killoran, Metzer, Nizic, Zhang and Russo conducted experiments. Capel, Coxon, D’Aloisio, Hutcheon, Kendall, Killoran, Metzer, Nizic and Kuburas performed data analysis. Coxon, Hutcheon, Kendall, Killoran, Metzer, Russo and Kuburas wrote or contributed to the writing of the manuscript.
